# M6229 Protects against Extracellular-Histone-Induced Liver Injury, Kidney Dysfunction, and Mortality in a Rat Model of Acute Hyperinflammation

**DOI:** 10.3390/ijms25031376

**Published:** 2024-01-23

**Authors:** Chris P. M. Reutelingsperger, Marion J. Gijbels, Henri Spronk, Rene Van Oerle, Roy Schrijver, Peter Ekhart, Sjef de Kimpe, Gerry A. F. Nicolaes

**Affiliations:** 1 Department of Biochemistry, Cardiovascular Research Institute Maastricht (CARIM), Maastricht University, 6200 MD Maastricht, The Netherlands; c.reutelingsperger@maastrichtuniversity.nl (C.P.M.R.); henri.spronk@maastrichtuniversity.nl (H.S.); rene.vanoerle@maastrichtuniversity.nl (R.V.O.); 2 Department of Pathology, Maastricht University Medical Center, MUMC+, 6202 AZ Maastricht, The Netherlands; m.gijbels@maastrichtuniversity.nl; 3 Department of Medical Biochemistry, Amsterdam Cardiovascular Sciences—Atherosclerosis & Ischemic Syndrome, Amsterdam Infection and Immunity—Inflammatory Diseases, Amsterdam UMC Location University of Amsterdam, Meibergdreef 9, 1105 AZ Amsterdam, The Netherlands; 4 Coagulation Profile B.V., 6229 EV Maastricht, The Netherlands; 5 Matisse Pharmaceuticals B.V., 6163 JT Geleen, The Netherlands; r.schrijver@matissepharmaceuticals.com (R.S.); p.ekhart@matissepharmaceuticals.com (P.E.); s.kimpe@matissepharmaceuticals.com (S.d.K.)

**Keywords:** M6229, sepsis, hyperinflammation, extracellular histones, DAMPs, organ injury, rat model

## Abstract

Extracellular histones have been shown to act as DAMPs in a variety of inflammatory diseases. Moreover, they have the ability to induce cell death. In this study, we show that M6229, a low-anticoagulant fraction of unfractionated heparin (UFH), rescues rats that were challenged by continuous infusion of calf thymus histones at a rate of 25 mg histones/kg/h. Histone infusion by itself induced hepatic and homeostatic dysfunction characterized by elevated activity of hepatic enzymes (ASAT and ALAT) and serum lactate levels as well as by a renal dysfunction, which contributed to the significantly increased mortality rate. M6229 was able to restore normal levels of both hepatic and renal parameters at 3 and 9 mg M6229/kg/h and prevented mortality of the animals. We conclude that M6229 is a promising therapeutic agent to treat histone-mediated disease.

## 1. Introduction

Histones, a family of proteins present in the nucleus of all nucleated eukaryotic cells, have important roles in gene expression and chromatin condensation. While they play a pivotal role in the nucleus, when present in the extracellular environment, extracellular histones have been found to possess cytotoxic properties in a variety of biological systems. Histones are released from the nucleus into the extracellular space through several different pathways and have been reported to be involved in numerous pathological processes, including inflammation, thrombosis, apoptosis, and prominently in sepsis. In recent years, it has become apparent that extracellular histones can exert their cytotoxic effects through direct and indirect pathways. In the first, histones have been described to directly influence mitochondrial and plasma membrane permeability and stability, which may lead to pore formation and cell lysis. Also, Ca^2+^ homeostasis is known to be disturbed in cells after exposure to histones, contributing to the direct toxic effects of extracellular histones.

The indirect pathways include histone-mediated activation through TLR receptor and the NOD2/NLRP3 pathways and the inhibition of complement. See a recent review [1] for a more complete overview of the molecular mechanisms involved in the expression of cytotoxicity by extracellular histones. Extracellular histones have been described to be toxic to cells of various origin, ranging from endothelial cells [2,3] to lung epithelial cells [4,5,6], liver cells [7,8], cardiac myocytes [9,10] and smooth muscle cells [11,12], renal endothelial and epithelial cells [13,14], and blood cells like thrombocytes [15,16] and erythrocytes [16,17,18].

Extracellular histones have likely evolved as a defense mechanism against pathogen infection, as part of the innate immune system, where due to their toxicity, histones are able to kill, e.g., an invading bacterium. This occurs most evidently in the process of neutrophil extracellular trap (NET) formation, also called NETosis [19]. Not only neutrophils are able to expel their nuclear content; other cell types have also been described to exhibit extracellular trap (ET) formation, such as macrophages and smooth muscle cells [20]. Notably, in NETs, the majority of proteins consist of histones [21]. Importantly, no matter the origin of an extracellular histone, it is an intrinsically toxic molecule that can cause cell death. The uninhibited exposure of extracellular histones to the blood is recognized as a major contributor to death in sepsis [2], which has spurred research into histone-mediated toxicity and into ways to prevent such toxicity by targeting the extracellular histones.

In this context, neutralization of the positively charged histones by negatively charged glycosaminoglycans, a group of biomolecules to which heparins also belong, was identified as a promising strategy to treat histone-mediated diseases [22,23]. Wildhagen and co-workers also demonstrated that the pentasaccharide motif, which mediates the anticoagulant activity of heparins [24], was not required for the histone-neutralizing activity of heparins [22]. Recently, the heparin molecules lacking the pentasaccharide motif, and, hence, anticoagulant activity, were isolated from unfractionated heparin (UFH) in a GMP procedure based on the principle of affinity chromatography as described by the group of Lindahl [25]. A Phase I/IIa clinical study has recently been completed on the obtained low-anticoagulant fraction of UFH, called M6229, as a therapeutic treatment for septic patients in intensive care.

In this paper, we report the first preclinical study of M6229 in a rat model of hyperinflammation induced by continuous infusion of extracellular histones. We show that infusion with M6229 is well tolerated and rescues rats from histone-induced liver injury, kidney dysfunction, and mortality.

## 2. Results

### 2.1. M6229 Has Low Anticoagulant Activity

**Figure 1:**
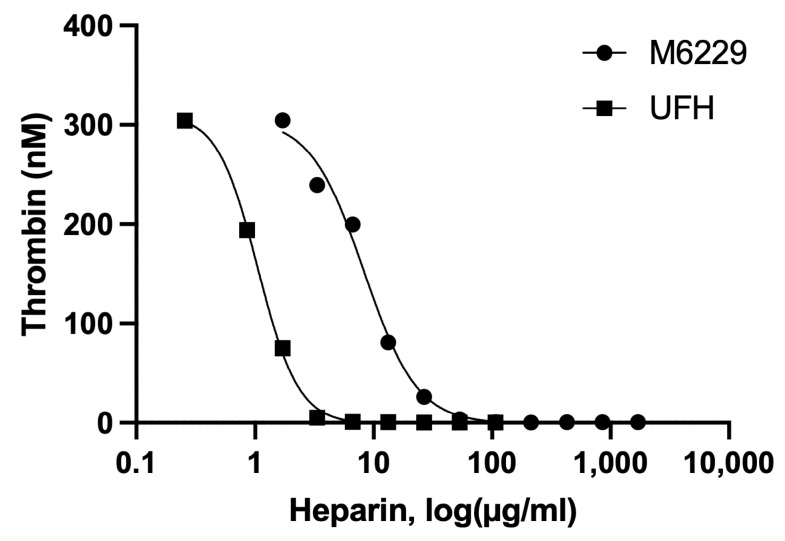
Dose–responses of UFH and M6229 doses on TG in citrated human plasma, triggered with PPP Reagent Int-High.

Affinity chromatography of UFH using immobilized antithrombin separates the heparin molecules containing the pentasaccharide from those that do not contain the antithrombin-binding motif that confers anticoagulant activity on the heparin molecule. When dose–responses were measured in the TG assay [26], the IC50 value of the latter fraction (M6229) was 16.5 µg/mL, whereas UFH had an IC50 value of 2.0 µg/mL (Figure 1).

### 2.2. M6229 Rescues Rats from Extracellular-Histone-Induced Mortality

**Figure 2:**
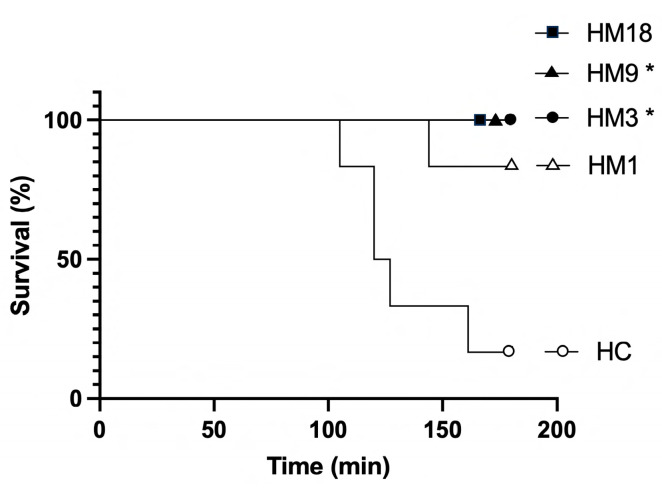
Kaplan–Meier curves of the survival of the rats of experimental groups HC, HM1, HM3, HM9, and HM18. * *p* < 0.01 for HM3 and HM9 vs. HC, as determined by the Chi-square and Fisher’s exact tests.

The rats were divided into nine experimental groups, as described in Table 1. Intravenous infusion of a histone mixture isolated from bovine thymus at a rate of 25 mg/kg/h caused the death of five out of six rats between 105 and 161 min after the start of the infusion. Starting the M6229 therapy at 60 min, HM3 and HM9 had significantly higher survival than HC (Figure 2). HM18 had no mortality, but the number of animals was too low to reach significance. All rats in the M1, M3, M9, and M18 groups survived the infusion of M6229.

### 2.3. Circulating M6229 Levels in Plasma of M and HM Rats

**Figure 3:**
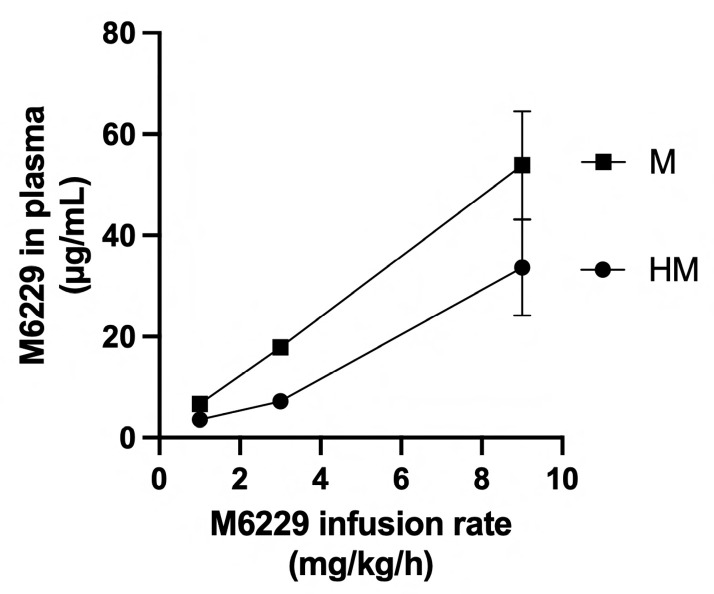
M6229 levels in plasmas of rats after 1 h infusion of M6229 at rates of 1 mg/kg/h, 3 mg/kg/h, and 9 mg/kg/h in the absence (M) or presence (HM) of histone infusion. Data points are mean ± S.D.

M6229 levels were measured in citrated plasma collected from the rats of M1 (*n* = 2), M3 (*n* = 2), M9 (*n* = 2), HM1 (*n* = 6), HM3 (*n* = 6), and HM9 (*n* = 4) 1 h after the start of histone infusion. An increase in the dose of infused M6229 led to an increase in M6229 in the plasma (Figure 3). Interestingly, the level of circulating M6229 was lower in the HM groups than in the M groups.

### 2.4. M6229 Infusion Prolongs aPTT but Not PT of Platelet-Poor Plasma from M and HM Rats

**Figure 4:**
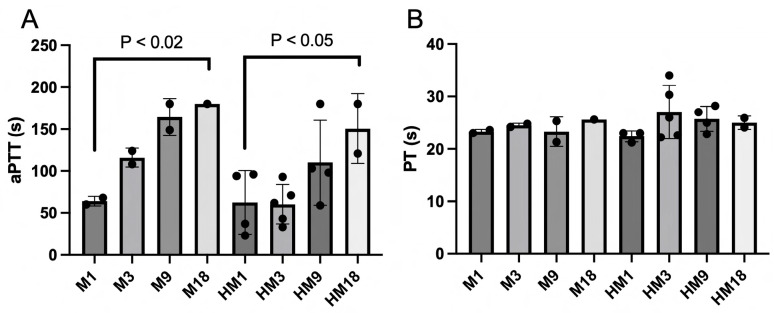
The coagulation times aPTT (panel (**A**)) and PT (panel (**B**)) were measured in PPP collected at 2 h after the start of M6229 infusion from M1 (*n* = 2), M3 (*n* = 2), M9 (*n* = 2), M18 (*n* = 1), HM1 (*n* = 4 panel (**A**), *n* = 3 panel (**B**)), HM3 (*n* = 5), HM9 (*n* = 4), and HM18 (*n* = 2). Statistically significant differences within M and within HM were determined with the Kruskal–Wallis test.

At 3 h after the start of histone infusion, the surviving animals were sacrificed and citrated blood was collected and processed into PPP. The coagulation times of the PPP were measured by aPTT and PT (Figure 4A,B). A significant increase in aPTTs was observed in the M and HM groups with increasing M6229 dose. PT measurement of the same samples did not reveal any differences in PT coagulation time.

### 2.5. Red Blood Cell and Platelet Count

**Figure 5:**
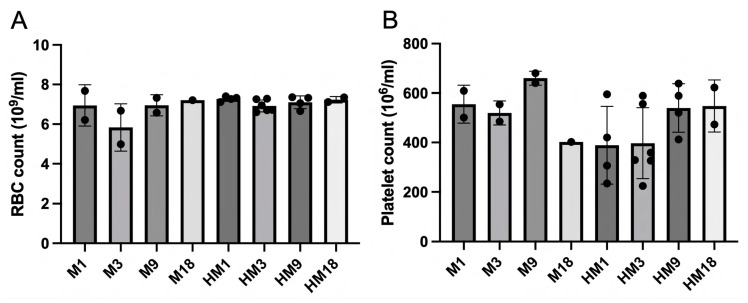
RBC (**A**) and platelet count (**B**) as determined in whole blood collected from M1 (*n* = 2), M3 (*n* = 2), M9 (*n* = 2), M18 (*n* = 1), HM1 (*n* = 4), HM3 (*n* = 6), HM 9 (*n* = 4), and HM18 (*n* = 2). No statistically significant differences between groups and within M and HM were observed with the Mann–Whitney and Kruskal–Wallis tests, respectively.

RBC and platelet count were determined at the termination of the experiment at 3 h of histone infusion. No statistically significant differences were present between the groups for these parameters (Figure 5).

### 2.6. M6229 Infusion Prevents Extracellular-Histone-Induced Liver Injury and Kidney Dysfunction

**Figure 6:**
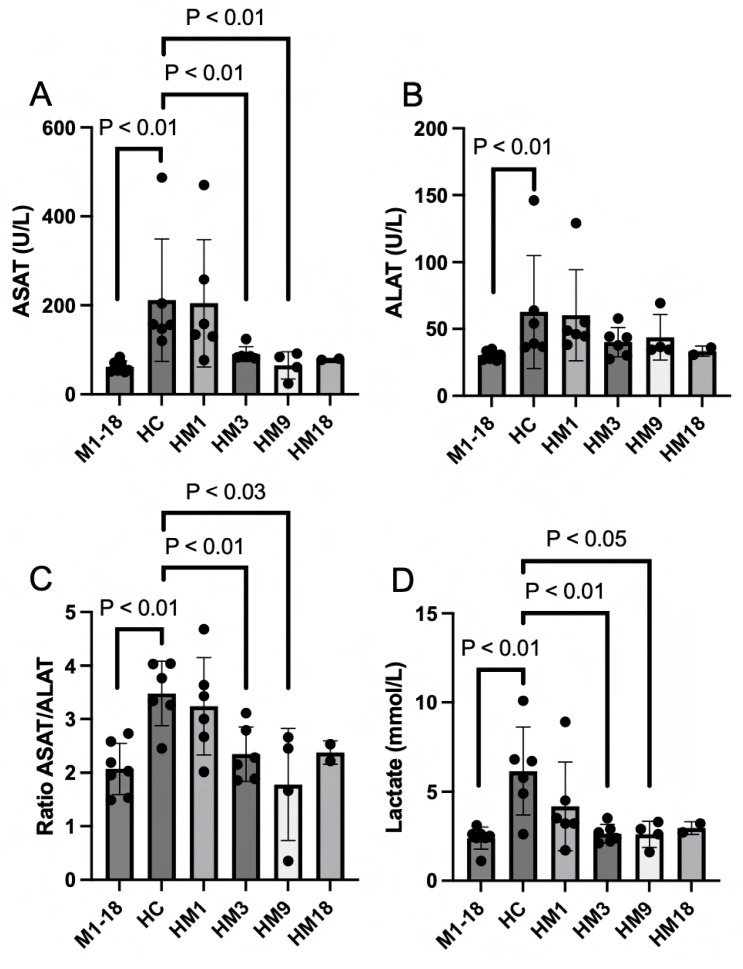
Circulating biomarkers ASAT (panel (**A**)), ALAT (panel (**B**)), and lactate (panel (**D**)) were measured in serum obtained from M1 (*n* = 2), M3 (*n* = 2), M9 (*n* = 2), M18 (*n* = 1) (collected in M1-18), HC (*n* = 6), HM1 (*n* = 6), HM3 (*n* = 6), HM9 (*n* = 4), and HM18 (*n* = 2) at termination of the experiment (at 3 h histone infusion or mortality). The ratio of ASAT/ALAT (panel (**C**)) was calculated for each group. Statistically significant differences between groups were determined with the Mann–Whitney test.

The circulating biomarkers ASAT (Figure 6A), ALAT (Figure 6B), and lactate (Figure 6D) were measured in the serum samples obtained at the termination of the experiment (at 3 h of histone infusion or at mortality). All three biomarkers and the calculated ASAT/ALAT ratio (Figure 6C) demonstrated that histone infusion causes liver injury with decreased liver function. Therapy with M6229 infusion 1 h after the start of the histone challenge significantly decreased liver injury and restored liver function with the HM3 and HM9 treatment regimens. The HM18 dose did not reach significance because of the *n* = 2.

**Figure 7:**
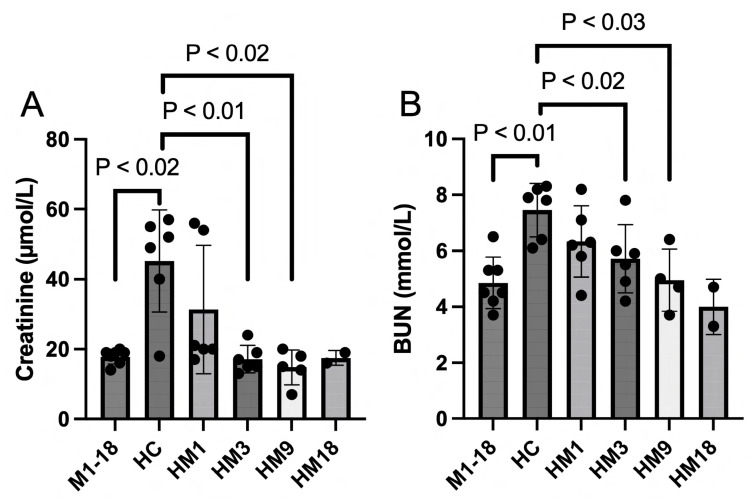
Circulating biomarkers creatinine (panel (**A**)) and BUN (panel (**B**)) were measured in serum obtained from M1 (*n* = 2), M3 (*n* = 2), M9 (*n* = 2), M18 (*n* = 1) (collected in M1-18), HC (*n* = 6), HM1 (*n* = 6), HM3 (*n* = 6), HM9 (*n* = 4), and HM18 (*n* = 2) at termination of the experiment (at 3 h histone infusion or mortality). Statistically significant differences between groups were determined with the Mann–Whitney test.

The circulating biomarkers creatinine (Figure 7A) and BUN (Figure 7B) were increased as a result of the histone infusion, indicating extracellular-histone-induced kidney dysfunction. Infusion with M6229 alone (M1-18) had no effect on kidney function. Therapy with M6229 infusion restored kidney function significantly in the HM3 and HM9 groups. The HM18 group did not reach significance because of *n* = 2.

### 2.7. Histopathology

**Figure 8:**
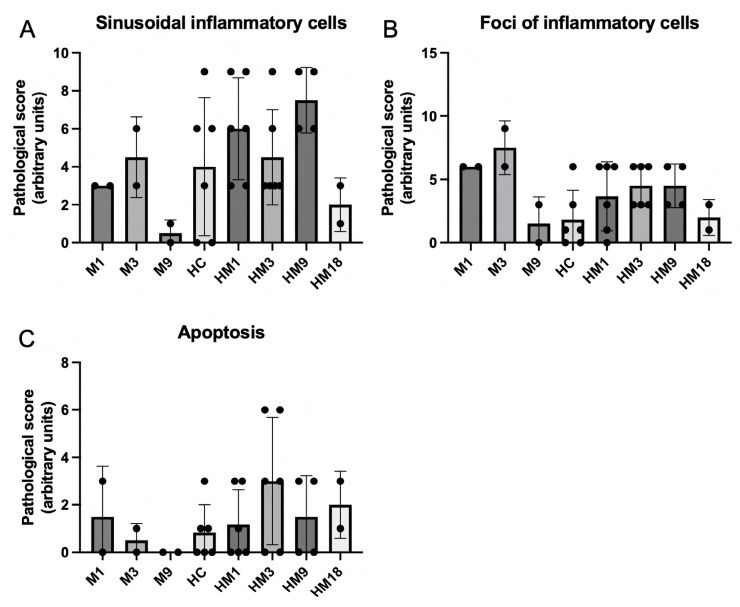
Histopathological analysis of the presence of sinusoidal cells (panel (**A**)), inflammatory cells (panel (**B**)), and apoptotic cells (panel (**C**)) of sections of liver obtained from M1 (*n* = 2), M3 (*n* = 2), M9 (*n* = 2), HC (*n* = 6), HM1 (*n* = 6), HM3 (*n* = 6), HM9 (*n* = 4), and HM18 (*n* = 2) at termination of the experiment (at 3 h histone infusion or mortality). The analyses were transformed into numerical scores as described.

Semi-quantification of the presence of sinusoidal inflammatory cells, the foci of inflammatory cells, and apoptotic cells in H&E-stained liver sections did not reveal statistically significant differences between the groups infused with histones and varying amounts of M6229 (Figure 8). Sinusoidal inflammatory cells, the foci of inflammatory cells, and apoptotic cells were also observed in the groups which were not infused with histones, suggesting that all animals had a low grade of liver inflammation that was not significantly aggravated by extracellular histones within the timeframe of their infusion.

Histopathological analysis of H&E-stained kidney sections revealed sporadic presence of protein-rich material in the tubuli of rats of all groups studied. The overall assessment by the pathologist was that the kidneys looked healthy, and no abnormalities were observed other than the presence of protein-rich material in some of the tubuli.

## 3. Discussion

The past decades have revealed a pathological role for extracellular histones in inflammatory diseases [27] including sepsis [2] and COVID-19 [1]. It has been shown that intravenously administered histones have a lethal effect in a variety of animal models [2,28]. They cause organ dysfunction through a plethora of cellular and humoral activities, which are believed to arise from their polycationic nature. Polyanionic compounds such as heparins can neutralize histones by complexation through electrostatic interactions [29]. About 30% of the molecules in UFH carry the pentasaccharide motif responsible for the anticoagulant activity of UFH [30]. Antithrombin-affinity chromatography of UFH yields the molecules that have low anticoagulant activity [25]. Wildhagen and co-workers showed that the low-anticoagulant fraction of UFH retains histone-neutralizing activity and rescues mice lethally challenged with cecal ligation puncture and LPS [22]. The low-anticoagulant heparin fraction has an advantage over UFH since in non-anticoagulant treatments it can be administered at higher doses. M6229 is the low-anticoagulant fraction of UFH that is in clinical development for the treatment of septic patients in the ICU. The current study assessed the therapeutic potential of M6229 in a rat model of acute and fatal inflammation induced by intravenous infusion of extracellular histones. M6229 exhibits residual anticoagulant activity as measured in a thrombin generation test with citrated human plasma. Its anticoagulant potency is about 8 times less than UFH and is probably caused by residual heparin molecules containing pentasaccharide sequences. A 2 h M6229 infusion into rats caused an M6229-dose-dependent increase in aPTT but no hemorrhagic complications. The PT was not significantly affected by M6229 infusion, as expected from the relative insensitivity of PT for heparins [31]. M6229 in the absence of histones had no significant effects on RBC and platelet count, nor did it raise the levels of serum ASAT, ALAT, lactate, creatinine, and BUN, indicating that the dosing regimens of M6229 were well tolerated.

Intravenous infusion of 25 mg histones/kg/h caused, in the absence of M6229, an acute and lethal response in five out of six rats within 3 h of the start of the infusion. At death, the serum levels of ASAT, ALAT, lactate, creatinine, and BUN were increased, which suggests the presence of histone-induced tissue damage, most likely of the liver and kidney. M6229 infusion into histone-treated rats 1 h after the start of the histone challenge dose-dependently prolonged the aPTT but not as much as in the unchallenged rats. The reduced anticoagulant activity in the histone-treated rats could be explained by an inhibition of M6229′s anticoagulant activity through binding of the circulating histones to M6229, similar to the reported inhibition of UFH’s anticoagulant activity in the presence of histones [32]. In the histone-challenged animals, administration of the M6229 resulted in a lowering of the levels of serum ASAT, ALAT, lactate, creatinine, and BUN to the levels of the control animals that had received M6229 only. This suggests that when complexed to M6229, the histone-associated cytotoxicity in vivo is abolished, in line with in vitro experiments that show that the complex of M6229 and histones does not induce cell death [22]. Histones bind RBCs and platelets, and their intravenous administration causes a dramatic drop in platelet count in mice without affecting RBC count [16]. We were not able to count platelets and RBCs in the 5 HC animals that died prematurely. The surviving HC animal had 7.43 × 10^9^ RBCs/mL and 402 × 10^6^ platelets/mL at sacrifice, indicating that the infused histones did not dramatically alter the platelet count in this model. M6229 infused at a rate of 3 mg/kg/h and higher completely prevented the lethal effect of the histones and alleviated histone-induced liver injury and kidney dysfunction as judged from the serum biochemistry. Microscopically, for the setup chosen here, the liver and kidney did not reveal significant differences within and between the experimental groups. Liver damage and kidney dysfunction as indicated by the serum biochemistry were likely the consequence of histone-induced perturbation of endothelial cells [2]. Endothelial cells are covered at their luminal side with an extracellular layer, the glycocalyx, of which glycosaminoglycans constitute the major part [33]. The glycocalyx forms a protective barrier to extracellular histones [34] and is likely disrupted as a consequence of histone challenge [35]. Hence, in the histone-challenged rats, the M6229 may provide protection by shielding endothelial cells and replenishing the disturbed glycocalyx barrier. It is noteworthy that M6229, unlike UFH, probably does not interfere with the antithrombin-mediated protection of the glycocalyx [36] because it lacks the pentasaccharide motif.

In conclusion, M6229 is a promising therapeutic agent to treat extracellular histone-mediated inflammatory diseases such as sepsis. M6229 is well tolerated and effectively blocks the organ-damaging and lethal effects of extracellular histones.

## 4. Materials and Methods

### 4.1. Histone Preparation

Purified calf thymus histones (Roche, Basel, Switzerland) were dissolved in PBS, pH 7.22–7.4 (using 3 cycles of 10 min sonication impulses at 40 °C), 1 day before the experimental day and kept at 4 °C until use. Protein concentration of the histone solution was determined at each experimental day, and the concentrations were used to set up histone infusion rates (which were kept below a 20 mL/kg/h volume load).

### 4.2. M6229 Preparation

M6229 was obtained from clinical GMP-grade UFH (Smithfield Bioscience, Cincinnati, OH, USA) based on affinity chromatography employing immobilized antithrombin, as described in [22]. M6229 lacks the heparin fraction that contains the specific pentasaccharide sequence that is required for the anticoagulant properties of UFH.

### 4.3. Thrombin Generation Assay

Thrombin generation (TG) was performed in platelet-poor citrated human plasma using the Calibrated Automated Thrombogram method, as previously described [26]. Briefly, to plasma, phospholipids were added in a 20/60/20 (Phosphatidylserine/Phosphatidylcholine/Phosphatidylethanolamine) molar ratio to the concentration of 4 μM. UFH (Smithfield Bioscience) and M6229 were added at the indicated concentrations. Thrombin generation was triggered by PPP Reagent Int-High (based on ellagic acid, reflecting the intrinsic route of coagulation activation, similar to aPTT) and measured using a low-affinity fluorescent thrombin substrate (Z-Gly-Gly-Arg 7-amino-4-methylcoumarin) (Thrombinoscope, Stago, Parsippany, NJ, USA) in a Fluoroskan Ascent reader (Thermo Fisher, Waltham, MA, USA) with the CAT control and evaluation software (version 5.0.0.742, Thrombinoscope, Stago).

### 4.4. Ethics Statement

All animal experiments were carried out according to the Guide for the Care and Use of Laboratory Animals DHEW (NIH) and Directive 2010/63/EU on the protection of animals used for scientific purposes, and the study was approved by the Animal Ethics Committee of Csongrád County Government Office, General Department of Public Health and Food Chain Safety, Department of Food Chain Safety and Animal Health, Hungary (approval number: XXVIII./455/2019).

### 4.5. Animals and Housing

Thirty-one healthy adult (8–11-week-old) male Wistar-Hannover rats (Toxicoop, Budapest, Hungary) weighing 313–408 g were included in the study. The animals were housed in individually ventilated cages using HEPA-filtered air (IVC system, Tecniplast, Buguggiate, Italy), which conformed to the size recommendations in the most recent Guide for the Care and Use of Laboratory Animals DHEW (NIH) and Directive 2010/63/EU on the protection of animals used for scientific purposes. Litter material placed beneath the cage was replaced at least two times a week. The animal room was temperature-controlled, had a 12 h light/dark cycle, and was kept clean and vermin-free. The animals were acclimatized to the housing facilities for at least 5 days prior to testing and received standard laboratory rat chow ad libitum and had access to filtered tap water ad libitum.

### 4.6. Induction of Acute Fulminant Inflammation and M6229 Treatment

**Figure 9:**
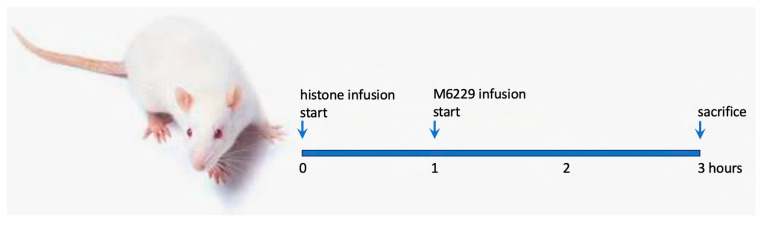
Schematic representation of the intravenous infusion regimen of histones and M6229 administration. Histones were infused at 25 mg/kg/h for a maximum of 3 h, and M6229 was administered at the doses 1, 3, 9, and 18 mg/kg/h for a maximum of 2 h. Surviving animals were sacrificed after 3 h of histone infusion.

After induction of anesthesia by pentobarbital sodium (Repose, 2.5%; 60 mg/kg, Le Vet Pharma, Oudewater, The Netherlands), both jugular veins were cannulated with a PE50 cannula to administer histones and M6229. Acute inflammation was induced by continuous intravenous infusion of histones in PBS (pH 7.22–7.40) at 25 mg/kg/h infusion rate (not exceeding 20 mL/kg/h volume load) for a maximum of 180 min by using a Perfusor compact S infusion pump (B. Braun Holding GmbH & Co., Melsungen AG, Germany). Animals were sacrificed 3 h after the start of histone infusion. Conventional 3-lead ECG (Einthoven I, II, III) was recorded throughout the experiments (Haemosys System, MDE Ltd., Budapest, Hungary). The right carotid artery was also cannulated to obtain arterial blood samples during the experiments. M6229 was administered at 1, 3, 9, or 18 mg/kg/h, starting at the 60th min of histone infusion as continuous infusion until termination of the animal (up to 120 min) by using a Perfusor compact S infusion pump (see Figure 9). Sterile physiological saline (0.9% NaCl) + 1 mM Citric Acid, pH = 6.5) was used to make the volume load equal in all experimental groups. The experimental groups are listed in Table 1.

### 4.7. Blood and Tissue Sampling during Experiments

Whole blood (350–400 µL) was collected from the abdominal aorta into tubes containing 3.2% sodium citrate at 2 h after the start of histone infusion and at termination of the experiment (at 3 h of histone infusion or at mortality). For hematological analyses, whole blood was collected from abdominal aorta into tubes containing K-EDTA and tubes containing Z serum clot activator at termination of the experiment. If an animal died prematurely, blood was also collected from the pectoral cavity. These blood samples were used solely to measure the biochemistry (aspartate aminotransferase (ASAT), alanine aminotransferase (ALAT), creatinine, and blood urea nitrogen (BUN)). Platelet poor plasma (PPP) was obtained from anticoagulated whole blood and serum from clotted blood by 15 min centrifugation at 2000× *g* at 4 °C. Aliquots of PPP and serum were snap-frozen in liquid nitrogen and stored at <−70 °C until use.

At termination of the experiment, one lung, one liver lobe, and 2 kidneys were harvested per animal, immersed in 4% formaldehyde fixative, stored for 24 h at room temperature, and subsequently processed for embedding in paraffin.

### 4.8. Plasma Coagulation

Citrated plasma was used to measure the routine coagulation parameters activated partial thromboplastin time (aPTT) and prothrombin time (PT).

### 4.9. Plasma M6229 Measurement

M6229 levels were measured in citrated plasma samples using the Heparin Red Kit (Redprobes UG, Münster, Germany). The protocol of the manufacturer was employed.

### 4.10. Platelet and Red Blood Cell Counts

Counts of platelets and red blood cells (RBCs) were measured in K-EDTA anticoagulated whole blood using a Hitachi Cobas 8000 automated modular blood analyzer system.

### 4.11. Serum Biochemistry

Parameters of liver and kidney function were determined from serum samples. These parameters included ASAT and ALAT activities as well as creatinine, BUN, bilirubin, and albumin levels and were measured employing a Hitachi Cobas 8000 automated modular blood analyzer system. Serum lactate levels were measured by using an AccuTrend Plus bedside System. Lactate measurements were performed in duplicates, and the average of two measurements was used for statistical evaluation.

### 4.12. Histopathology

The paraffin-embedded tissues were cut into 10 sections (5 µm thick) per tissue sample (31 liver lobes and 62 kidneys). The sections were deparaffinized and stained with hematoxylin–eosin stain (H&E) for microscopical analysis. To facilitate quantitation of histopathological data, observational data were transformed to numerical data, following a 5-point scale, where absence of an observation (sinusoidal inflammatory cells, foci of inflammatory cells, and apoptosis) received a value of zero. Cases with doubt about the presence of the observations received a value of 1. Cases with the observation present, clearly present, and very clearly present received values of 3, 6, and 9, respectively. Sections were scored blindly by a trained animal pathologist.

### 4.13. Statistics

Data were analyzed for statistically significant changes between groups with different M6229 doses and within M and HM groups using the Mann–Whitney *U* test and the Kruskal–Wallis test, respectively, using Graphpad Prism 10 software. A significant difference in survival between the experimental groups was assessed using Chi-square test followed by Fisher’s exact test.

## Figures and Tables

**Table 1:** Overview of the experimental groups.

Experimental Group	Infusion Histones (mg/kg/h) Start Infusion on T = 0	Infusion M6229 (mg/kg/h) Start Infusion on T = 60 min	n at Start
HC	25	0	6
HM1	25	1	6
HM3	25	3	6
HM9	25	9	4
HM18	25	18	2
M1	0	1	2
M3	0	3	2
M9	0	9	2
M18	0	18	1

## Data Availability

The data presented in this study are available on request from the corresponding author. The data are not publicly available due to the stage of development and related data protection for M6229. Patent applications are under review.
